# Supplementary material for: The Dietary Inflammatory Index and Chronic Lymphocytic Leukaemia in the MCC Spain Study
Source: Nutrients. 2019 Dec 23;12(1):48. doi: 10.3390/nu12010048 (PMC7019557; doi:10.3390/nu12010048)
Supplement: Supplementary file 1 [file nutrients-12-00048-s001.pdf]

nutrients

Article

THE DIETARY INFLAMMATORY INDEX AND CHRONIC LYMPHOCYTIC LEUKAEMIA IN THE MCC-SPAIN STUDY

José Carlos Flores [1], Esther Gracia-Lavedan [1,2,3], Yolanda Benavente [2,4], Pilar Amiano [2,5], Dora Romaguera [3,6,7], Laura Costas [4], Claudia Robles [8], Eva Gonzalez-Barca [9], Esmeralda de la Banda [10], Esther Alonso [10], Marta Aymerich [11], Elias Campo [11,12], Trinidad Dierssen-Sotos [2,13], Rafael Marcos-Gragera [2,14,15], Marta María Rodriguez-Suarez [16,17,18], Marta Solans [2,14,15], Eva Gimeno [19], Paloma Garcia Martin [20], Nuria Aragones [2,21], Nitin Shivappa [22,23,24], James R. Hébert [22,23,24], Marina Pollan [2,25], Manolis Kogevinas [1,2,3,26], Silvia de Sanjose [2,4,27], Gemma Castaño-Vinyals [1,2,3,26,*,†] and Delphine Casabonne [2,4,*,†]

**** shared senior authorship**

1 Universitat Pompeu Fabra, Barcelona,08002, Spain; josecarlosfl@outlook.com (J.C.F.); esther.gracia@isglobal.org (E.G.-L.); manolis.kogevinas@isglobal.org (M.K.)
2 Consortium for Biomedical Research in Epidemiology and Public Health (CIBERESP), Madrid, 28029, Spain; ybenavente@iconcologia.net (Y.B.); epicss-san@euskadi.eus (P.A.); trinidad.dierssen@unican.es (T.D.-S.); rafael.marcos@udg.edu (R.M.-G.); martasolans@gmail.com (M.S.); nuria.aragones@salud.madrid.org (N.A.); mpollan@isciii.es (M.P.); sdesanjose@path.org (S.d.S.)
3 Instituto de Salud Global de Barcelona (ISGlobal), Barcelona, 08003 ,Spain; dora.romaguera@isglobal.org
4 Unit of Molecular Epidemiology and Genetic in Infections and Cancer (UNIC-Molecular), Cancer Epidemiology Research Programme (IDIBELL), Catalan Institute of Oncology, L'Hospitalet de Llobregat, 08908 Spain; lcostas@iconcologia.net
5 Public Health Division of Gipuzkoa, BioDonostia Research Institute, San Sebastian, 20014,Spain
6 Instituto de Investigación Sanitaria Illes Balears (IdISBa), Palma, 07120, Spain.
7 CIBER Fisiopatología de la Obesidad y Nutrición (CIBEROBN), Madrid, 28029, Spain.
8 Unit of Information and Interventions in Infections and Cancer (UNIC-I&I), Cancer Epidemiology Research Programme, (IDIBELL), Catalan Institute of Oncology, L'Hospitalet de Llobregat, 08908, Spain; crobles@idibell.cat
9 Haematology, Bellvitge Biomedical Research Institute (IDIBELL), Catalan Institute of Oncology, L'Hospitalet de Llobregat, 08908, Spain; e.gonzalez@iconcologia.net
10 Haematology Laboratory, Department of Pathology, Hospital Universitari de Bellvitge, L'Hospitalet de Llobregat, 08908, Spain; edelabanda@bellvitgehospital.cat (E.d.l.B.); ealonso@bellvitgehospital.cat (E.A.)
11 Hematopathology Unit, Department of Pathology, Hospital Clínic, (IDIBAPS), Barcelona, 08036, Spain; aymerich@clinic.cat (M.A.); ECAMPO@clinic.cat (E.C.)
12 Centro de Investigación Biomédica en Red Cáncer (CIBERONC), Instituto de Salud Carlos III (ISCIII), Madrid, 28029,Spain
13 University of Cantabria – Marqués de Valdecilla Research Institute (IDIVAL), Santander, 39011, Spain.
14 Research group on Statistics, Econometrics and Health (GRECS), University of Girona, Girona, 17071, Spain.
15 Epidemiology Unit and Girona Cancer Registry, Catalan Institute of Oncology, Girona, 17007, Spain.
16 Universidad de Oviedo, área de medicina Preventiva y Salud Pública, Oviedo, 33003, Spain; mrstsf@gmail.com
17 Hospital Universitario Central de Asturias (HUCA), Oviedo, 33011, Spain
18 IUOPA: Instituto de Oncología de Asturias (IUOPA), Oviedo, 33003, Spain.
19 Haematology Department, Hospital del Mar, Barcelona, 08003, Spain; 94015@parcdesalutmar.cat
20 Unidad de Gestión Clínica de Hematología. Hospital Universitario San Cecilio PTS de Granada, Granada, 18016, Spain; paloma.garcia.martin.sspa@juntadeandalucia.es
21 Epidemiology Section, Public Health Division, Department of Health of Madrid, Madrid,28035, Spain.
22 Cancer Prevention and Control Program, University of South Carolina, Columbia, SC 29208, USA; shivappa@mailbox.sc.edu (N.S.); jhebert@mailbox.sc.edu (J.R.H.)
23 Department of Epidemiology and Biostatistics, Arnold School of Public Health, University of South Carolina, Columbia, SC 29208, USA.
24 Connecting Health Innovations LLC (CHI), Columbia, SC 29201, USA.
25 Cancer Epidemiology Unit, National Centre for Epidemiology, Carlos III Institute of Health, Madrid, 28029, Spain.
26 Hospital del Mar Medical Research Institute (IMIM), Barcelona, 08003, Spain.
27 PATH, Sexual and Reproductive Health, Seattle, WA 98121, USA.

* Correspondence: dcasabonne@iconcologia.net; Tel.: (+34) 93 260 78 12

nutrients

Figure S1: Directed Acyclic Graph to evaluate the effect of potential confounders.

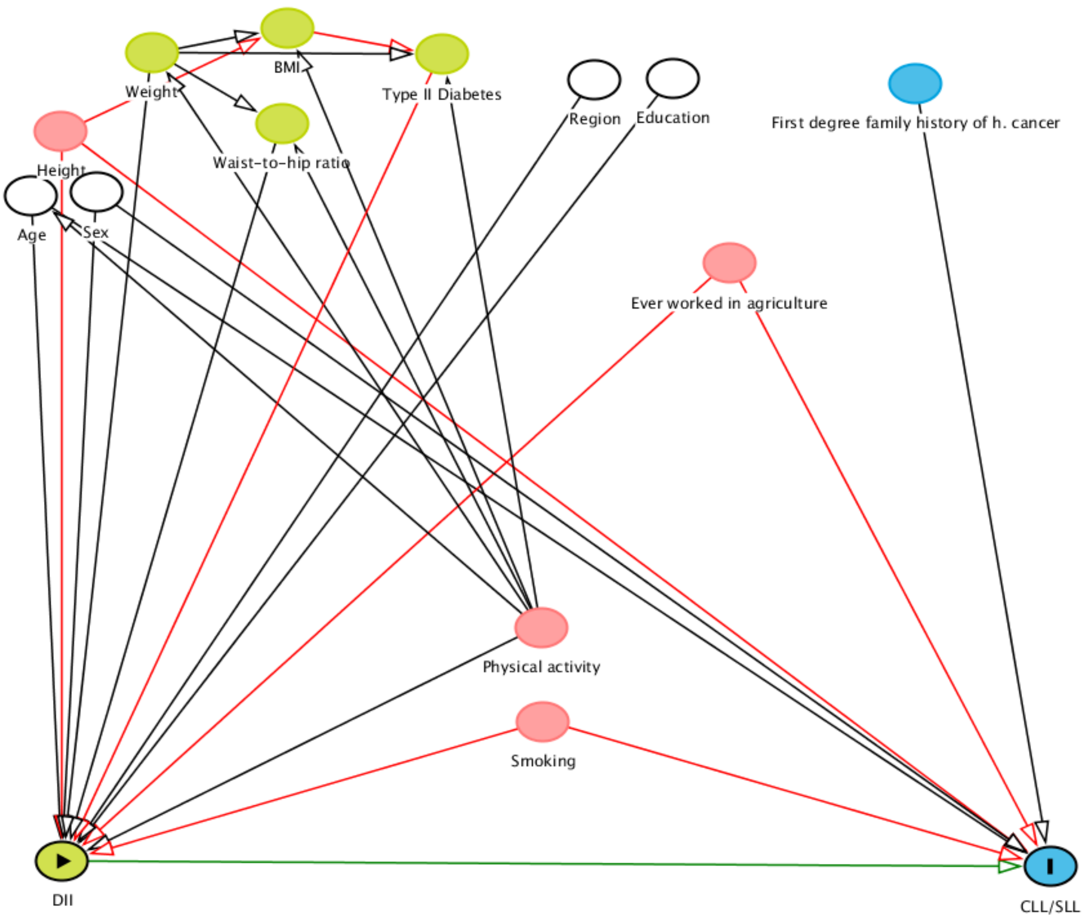

nutrients

Table S1: Evaluation of confounders: Odds ratios and 95% confidence interval of CLL for 1-unit increase in E-DII.

Model**	N in models Controls/cases	E-DII OR & 95% CI T1	T2	T3	*p-value* trend[a]	1-unit increase in E-DII OR & 95% CI	*p value for trend*	% of change in OR
Basic[b]	1643/366	Ref	1.20 (0.90, 1.59)	1.21 (0.90, 1.62)	0.21	1.05 (0.99, 1.12)	0.09	
Basic + first degree family history of haematological cancer	1535/351	Ref	1.14 (0.85, 1.53)	1.14 (0.84, 1.55)	0.39	1.04 (0.98, 1.11)	0.20	
Basic + ever worked in agriculture	1640/365	Ref	1.22 (0.92, 1.62)	1.25 (0.93, 1.69)	0.14	1.06 (1.00, 1.13)	**0.05**	
Basic + BMI	1643/366	Ref	1.20 (0.91, 1.60)	1.21 (0.90, 1.62)	0.21	1.06 (0.99, 1.12)	0.08	
Basic + weight	1621/365	Ref	1.18 (0.89, 1.57)	1.18 (0.87, 1.58)	0.28	1.05 (0.99, 1.12)	0.11	
Basic+ height	1591/354	Ref	1.22 (0.92, 1.63)	1.22 (0.90, 1.65)	0.20	1.06 (0.99, 1.12)	0.09	
Basic + alcohol consumption	1643/366	Ref	1.22 (0.92, 1.61)	1.27 (0.94, 1.71)	0.12	1.07 (1.00, 1.13)	**0.04**	
Basic + diabetes	1622/360	Ref	1.18 (0.89, 1.57)	1.19 (0.88, 1.61)	0.25	1.05 (0.99, 1.12)	0.10	
Basic + smoking	1638/364	Ref	1.21 (0.91, 1.60)	1.21 (0.89, 1.63)	0.21	1.06 (0.99, 1.12)	0.09	
Basic + energy	1643/366	Ref	1.18 (0.89, 1.56)	1.15 (0.85, 1.56)	0.35	1.05 (0.98, 1.11)	0.17	
Basic + waist-to-hip ratio	1629/364	Ref	1.20 (0.90, 1.59)	1.19 (0.88, 1.60)	0.26	1.05 (0.99 1.12)	0.12	
Basic+ physical activity	1605/353	Ref	1.17 (0.88, 1.57)	1.24 (0.91, 1.67)	0.17	1.06 (1.00, 1.13)	0.07	
Fully adjusted (based on DAG)[c]	1586/352	Ref	1.24 (0.93, 1.66)	1.24 (0.91, 1.68)	0.17	1.06 (1.00, 1.13)	0.07	
Basic[d]	1586/352	Ref	1.23 (0.92, 1.64)	1.21 (0.90, 1.64)	0.21	1.06 (0.99, 1.12)	0.09	0%

E-DII: Energy-adjusted Dietary Inflammatory Index, OR: Odds Ratio, 95% CI: 95% Confidence Interval, T: Tertile. DAG. Directed Acyclic Graph. BMI. Body mass index.
In bold: $p \leq 0.05$.
**Missing values for controls/cases: weight [22 (1.3%)/1 (0.27%)]; height [52 (3.2%)/12 (3.3%)]; waist-to-hip ratio 14 (0.9%) /2 (0.6%)]; Diabetes [21 (1.3%)/ 6 (1.6%)]; smoking [5 (0.3%)/ 2(0.6%)]; physical activity [38 (2.3%), 13 (3.6%)]; ever worked in agriculture [3 (0.2%)/ 1 (0.3%)]; first degree family history haematological cancer [108 (6.6%)/ 15 (4%)].
**Details of the variables: BMI(continuous), weight (continuous), height (continuous), alcohol consumption at time of interview (continuous), smoking (never, former, current), energy (continuous), waist-to-hip ratio (continuous), physical activity (inactive, low, moderate, very active).
[a]*p* trend for linear trend test (categories 1, 2, 3).
[b]Basic model is E-DII adjusted for sex, age, education (primary, secondary, university) and region (Barcelona region vs. other regions).
[c]Basic model adjusted for: height (continuous); smoking (never, former, current), ever worked in agriculture.
[d]Basic model is E-DII adjusted for sex, age, education (primary, secondary, university) and region (Barcelona region vs. other regions). Complete case analysis based to non-missing fully adjusted models.

nutrients

Figure S2 Distribution of key characteristics of CLL's controls according to DII in the MCC-Spain study.

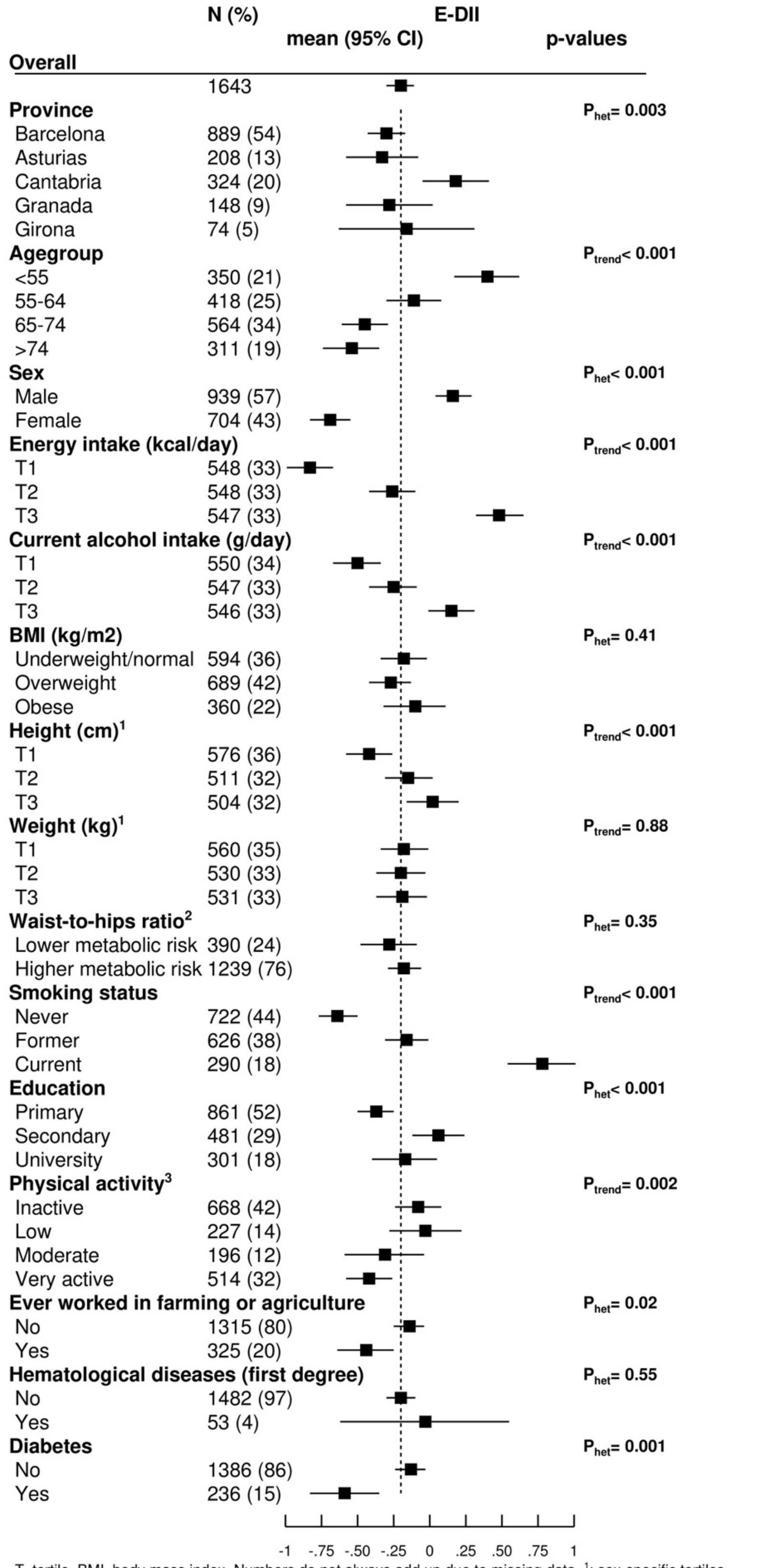

T, tertile, BMI, body mass index. Numbers do not always add up due to missing data. [1]: sex-specific tertiles. [2]: risk categories according to WHO criteria (0.90 for males and 0.85 for females).[3]: Physical activity, in the last 10 years, measured in METs/week: inactive (0), low (0.1-7.9), moderate (8-15.9) and very active (16 or more). Tertile cut-offs: energy in kcal/day (1590.02; 2077.0); alcohol in gr/day (1.41, 11.42); height in cm [168/158; 173/164, for men/women respectively]; weight in kg [74/61; 83/71, for men/women respectively].

nutrients

Table S2: Interaction analysis: Odds ratios and 95% confidence interval of CLL for 1-unit increase in E-DII

	N (control/CLL)	OR[a] (CI 95%)	*p* value
Overall	1643/366	1.05 (0.99, 1.12)	0.09
**Age (years)**			
<55	401 (350/51)	1.05 (0.90, 1.21)	0.54
55-64	515 (418/97)	1.03 (0.91, 1.16)	0.67
65-74	699 (564/135)	1.04 (0.94, 1.15)	0.47
≥75	394 (311/83)	1.06 (0.92, 1.22)	0.44
***p* interaction (multiplicative)**	0.72		
**RERI (CI 95%), *p* interaction**	0.51 (-0.03, 1.06), 0.06		
**AP (CI 95%), *p* interaction**	0.23 (-0.01, 0.47), 0.06		
**S (CI 95%), *p* interaction**	1.71 (0.79 to 3.71), 0.18		
**Sex**			
Male	1154 (939/215)	1.04 (0.96, 1.13)	0.32
Female	855 (704/151)	1.05 (0.95, 1.16)	0.31
***p* interaction (multiplicative)**	0.54		
**RERI (CI 95%), *p* interaction**	0.22 (-0.84, 0.40), 0.49		
**AP (CI 95%), *p* interaction**	-0.12 (-0.46, 0.22), 0.49		
**S (CI 95%), *p* interaction**	0.79 (0.43, 1.46), 0.46		
**Alcohol consumption**			
T1	689 (550/139)	1.04 (0.95, 1.15)	0.4
T2	679 (547/132)	1.02 (0.92, 1.13)	0.74
T3	641 (546/95)	1.13 (1.00, 1.27)	**0.05**
***p* interaction (multiplicative)**	0.25		
**RERI (CI 95%), *p* interaction**	0.19 (-0.19, 0.57), 0.33		
**AP (CI 95%), *p* interaction**	0.25 (-0.25, 0.75), 0.32		
**S (CI 95%), *p* interaction**	0.57 (NE), NE		
**Height**			
T1	702 (576/126)	1.04 (0.94, 1.16)	0.45
T2	625 (511/114)	1.02 (0.90, 1.14)	0.8
T3	618 (504/114)	1.08 (0.97, 1.20)	0.14
***p* interaction (multiplicative)**	0.13		
**RERI (CI 95%), *p* interaction**	0.24 (-0.26, 0.74), 0.35		
**AP (CI 95%), *p* interaction**	0.14 (-0.15, 0.43), 0.35		
**S (CI 95%), *p* interaction**	1.52 (0.50, 4.57), 0.46		
**Smoking**			
Never	886 (722/164)	1.13 (1.02, 1.25)	**0.02**
Former smoker	766 (626/140)	1.01 (0.91, 1.12)	0.84
Current smoker	350 (290/60)	0.97 (0.84, 1.12)	0.67
***p* interaction (multiplicative)**	0.41		
**RERI (CI 95%), *p* interaction**	-0.33 (-0.92, 0.26), 0.27		
**AP (CI 95%), *p* interaction**	-0.21 (-0.58, 0.16), 0.26		
**S (CI 95%), *p* interaction**	0.64 (0.33, 1.25), 0.19		
**Ever worked in agriculture**			
No	1573 (1315/258)	1.05 (0.98, 1.13)	0.19
Yes	432 (325/107)	1.08 (0.95, 1.23)	0.23
***p* interaction**	0.71		
**RERI (CI 95%), *p* interaction**	0.67 (-0.22, 1.56), 0.14		
**AP (CI 95%), *p* interaction**	0.23 (-0.04, 0.50), 0.10		
**S (CI 95%), *p* interaction**	1.54 (0.84, 2.81), 0.16		

OR: Odds Ratio, 95% CI: 95% Confidence Interval. p interaction: p value for interaction.
In bold p ≤ 0.05
*Additive interactions: RERI: relative excess risk due to interaction; AP: attributable proportion; S: synergy index; Categories for each variable: age (<64/65+); height [sex-median cut-offs (160cm for female, 170cm for male)]; agriculture (no/yes); smoking (never/ever); alcohol (low-medium/high). NE: not estimated. Models adjusted for age, sex and region.
[a]Basic model is E-DII adjusted for sex, age, education (primary, secondary, university) and region (Barcelona region vs. other regions).

nutrients

List S1: List of collaborators

MCC-SPAIN STUDY

Institut Català d'Oncologia (ICO)-L'Hospitalet de Llobregat
Víctor Moreno, Paloma Quesada, Yasmin Sabater, Marleny Vergara, Ainara Exposito, Teresa Alonso, Isabel Padrol, Joellen Klaustermeier, Yolanda Florencia, Vanesa Camon, Eva Domingo-Domenech, Dolores Dot, Anna Esteban, Elisabeth Guinó, Santi Mercadal, Ana Oliveira, Josep Sarrà.

ISGlobal- Centre for Research in Environmental Epidemiolog (CREAL), Barcelona, Spain
Mireia García, Cecília Persavento, Esther Gracia-Lavedan

Hospital Clínic, Barcelona, Spain
Cristina Capdevila Lozar, Ainara Expósito, Silvia Martin Román, Amparo Muñoz, Yolanda Torralba

Hospital del Mar, Barcelona, Spain
Eugènia Abella, Estela Carrasco, Judith Cirac, Francesc Garcia, Antonio Salar

Institut de Prestacions d'Assistència Mèdica al Personal Municipal (PAMEM) & Centros de Atención Primaria (CAP), Barcelona, Spain
Jesús Almeda, Marifé Alvarez Rodriguez, Alex Bassa Massanas, Albert Boada Valmaseda, Enric Duran, Olga Gonzalez Ferrer, Clara Izard, Manoli Liceran, Carmen López, Josep Manuel Benítez, Dolors Petitbó, Angelina Potrony, Sònia Sarret, Laura Sebastián, Josep M. Vilaseca

Instituto Universitario de Oncología, Universidad de Oviedo, Asturias, Spain
Cristina Arias, Guillermo Fernández-Tardón, Ana Fernández Somoano, Miguel García-Villarino, Carlos López-Otín,

Servicio de Salud del Principado de Asturias, SESPA, Asturias, Spain
Enrique Colado, Begoña Martínez-Argüelles, Manuel Rivas del Fresno, Marta María Rodríguez-Suárez.

Girona group
Unitat d'Epidemiologia i Registre de Cancer de Girona: Loreto Vilardell, Montse Puig-Vives, Gemma Osca-Gelis, M Carme Carmona-Garcia, Rocio Rodriguez Romanos, Carlota Torner Galindo, Patricia Martí Bargalló, Esther Rodriguez Sanchez, Marta Solans Margalef, Aina Roca Barceló, Raquel Comas Navarro, Marc Saez
Hospital Universitari de Girona Dr. Josep Trueta: Josep Maria Roncero, David Gallardo, Rosa Coll, Ignacio Blanco. *CAP de Santa Clara:* Conxa Bou
CAP de Angles: Gabriel Coll de Tuero, Alba Coll Negre

Granada group
University of Granada: Olmedo-Requena R, Olvera Porcel MC, Salcedo-Bellido I, Barrios-Rodríguez R, Jiménez-Moleón JJ;

Virgen de las Nieves,Universitary Hospital,Unit of Hematology: García-Martín P, Jurado-Chacón M.

Cantabria group

Inés Gómez-Acebo, Pilar González Echezarreta, Maria del Mar González Martínez, Luis Mariano López López, Almudena de la Pedraja Pavón, Paula Picón Sedano, Trinidad Dierssen-Sotos.

Gipuzkoa-MCC group & Institutions

Pilar Amiano, Madalen Oribe, Mikel Azpiri, Ana Jimenez, Carmen Urtiaga, M. Jesús Mitxelena, Concepcion Mugarza, Irune Ruiz

Public Health Division of Gipuzkoa, Hospital Universitario Nª Sra de Aranzazu, Basque Health Department; Onkologikoa, Instituto Oncologico de Gipuzkoa.
